# Case-control study of the association between malignant brain tumours diagnosed between 2007 and 2009 and mobile and cordless phone use

**DOI:** 10.3892/ijo.2013.2111

**Published:** 2013-09-24

**Authors:** LENNART HARDELL, MICHAEL CARLBERG, FREDRIK SÖDERQVIST, KJELL HANSSON MILD

**Affiliations:** 1 Department of Oncology, University Hospital, SE-701 85 Örebro, Sweden;; 2 Department of Radiation Sciences, Umeå University, SE-901 87 Umeå, Sweden

**Keywords:** ipsilateral, 25-years latency, time since first exposure, glioma, wireless phones

## Abstract

Previous studies have shown a consistent association between long-term use of mobile and cordless phones and glioma and acoustic neuroma, but not for meningioma. When used these phones emit radiofrequency electromagnetic fields (RF-EMFs) and the brain is the main target organ for the hand-held phone. The International Agency for Research on Cancer (IARC) classified in May, 2011 RF-EMF as a group 2B, i.e. a ‘possible’ human carcinogen. The aim of this study was to further explore the relationship between especially long-term (>10 years) use of wireless phones and the development of malignant brain tumours. We conducted a new case-control study of brain tumour cases of both genders aged 18–75 years and diagnosed during 2007–2009. One population-based control matched on gender and age (within 5 years) was used to each case. Here, we report on malignant cases including all available controls. Exposures on e.g. use of mobile phones and cordless phones were assessed by a self-administered questionnaire. Unconditional logistic regression analysis was performed, adjusting for age, gender, year of diagnosis and socio-economic index using the whole control sample. Of the cases with a malignant brain tumour, 87% (n=593) participated, and 85% (n=1,368) of controls in the whole study answered the questionnaire. The odds ratio (OR) for mobile phone use of the analogue type was 1.8, 95% confidence interval (CI)=1.04–3.3, increasing with >25 years of latency (time since first exposure) to an OR=3.3, 95% CI=1.6–6.9. Digital 2G mobile phone use rendered an OR=1.6, 95% CI=0.996–2.7, increasing with latency >15–20 years to an OR=2.1, 95% CI=1.2–3.6. The results for cordless phone use were OR=1.7, 95% CI=1.1–2.9, and, for latency of 15–20 years, the OR=2.1, 95% CI=1.2–3.8. Few participants had used a cordless phone for >20–25 years. Digital type of wireless phones (2G and 3G mobile phones, cordless phones) gave increased risk with latency >1–5 years, then a lower risk in the following latency groups, but again increasing risk with latency >15–20 years. Ipsilateral use resulted in a higher risk than contralateral mobile and cordless phone use. Higher ORs were calculated for tumours in the temporal and overlapping lobes. Using the meningioma cases in the same study as reference entity gave somewhat higher ORs indicating that the results were unlikely to be explained by recall or observational bias. This study confirmed previous results of an association between mobile and cordless phone use and malignant brain tumours. These findings provide support for the hypothesis that RF-EMFs play a role both in the initiation and promotion stages of carcinogenesis.

## Introduction

In May, 2011, the International Agency for Research on Cancer (IARC) at WHO evaluated the carcinogenic effect to humans from radiofrequency electromagnetic fields (RF-EMF). It included radiation from mobile phones, and from other devices that emit similar non-ionising electromagnetic fields. It was concluded that RF-EMF is a group 2B, i.e. a ‘possible’ human carcinogen (1,2).

The IARC evaluation of mobile phones was based mainly on case-control studies from the Hardell group in Sweden and the IARC Interphone study. Both sets of studies provided corroborative results, demonstrating an association between two types of brain tumours, glioma and acoustic neuroma, with exposure to RF-EMF from wireless phones. There was no consistent pattern of an association within the studied latency period (time since first exposure) with the most common benign brain tumour, meningioma, suggesting specificity for these other tumour types. However, it should be noted that in Interphone a reduced risk was found for glioma among regular users of mobile phones but an increased risk was found in the highest cumulative exposure group, >1,640 h (3). Clearly an increased risk was found using 1–1.9 years as reference entity (data not shown). The pros and cons in the Interphone study have been discussed in several articles, e.g. Hardell *et al* (4,5), Cardis and Sadetzki (6).

We first provide some background to the development of the wireless technology because of its relevance to understanding the nature of exposures and exposure assessments.

The Nordic countries were among the first countries in the world to widely adopt wireless telecommunications technology. Analogue phones (NMT, Nordic Mobile Telephone System) were introduced in the early 1980s using both 450 and 900 Megahertz (MHz) frequencies. NMT 450 was used in Sweden from 1981, but closed down on 31 December, 2007; NMT 900 operated during 1986–2000.

The digital system (GSM, Global System for Mobile Communication) using dual band, 900 and 1,800 MHz, started to operate in 1991, and it now dominates the market. The third generation of mobile phones, 3G or UMTS (Universal Mobile Telecommunication System), using 1,900/2,100 MHz RF fields has been introduced worldwide in recent years, and in Sweden in 2003. Currently, the fourth generation, 4G (Terrestrial 3G), operating at 800/2,600 MHz, and Trunked Radio Communication (TETRA 380–400 MHz) are being established in Sweden and elsewhere in Europe. Nowadays mobile phones are used more than landline phones in Sweden (http://www.pts.se/upload/Rapporter/Tele/2011/sv-telemarknad-halvar-2011-pts-er-2011-21.pdf). Worldwide, an estimate of 5.9 billion mobile phone subscriptions was reported at the end of 2011 by the International Telecommunication Union (http://www.itu.int/ITU-D/ict/facts/2011/material/ICTFactsFigures2011.pdf).

Desktop cordless phones (DECT) have been used in Sweden since 1988, first using analogue 800–900 MHz RF fields, but since the early 1990s using a digital 1,900 MHz system. They are very common, overtaking phones connected to landlines. Also, these devices emit RF-EMF radiation when used and should be equally considered as mobile phones when human health risks are evaluated.

The old analogue phones in Sweden, the so called NMT, had an output power of 1 W and were very seldom down-regulated giving lower RF-EMF emissions when used since the distance between the base stations was several kilometers. The GSM phones are transmitting in a pulsed mode, active 1/8 of the time, and with a maximum output power of 2 W. This could be downregulated depending on the distance to the base stations. A typical mean value for the average output power is around 50–60 mW. The phone always starts the call with the maximum power before going down in power. The digital cordless phones operate in pulsed mode with a duty cycle of 1/24, the peak power is 250 mW. It is only the newer models that have regulation of the output power. The old ones always stayed with peak 250 mW, giving a time average of about 10 mW.

The absorption pattern, i.e. SAR values, associated with the phones is very different between different phones; some can give the peak value above the ear, some on the ear, and some even below the ear, see for instance Wilén *et al* (7). There are no known measurements of SAR for the cordless phones.

The first indication of an increased risk for brain tumours associated with the use of mobile phones was published more than 10 years ago (8). For tumours located in the temporal, occipital or temporoparietal lobe areas of the brain, an increased risk was found for ipsilateral mobile phone use. Exposure to radiation from wireless phones (mobile and cordless) is generally highest in the part of the brain that is near to the ear, the temporal lobe, on the same side of the head as the phone is generally held, ipsilateral exposure (9).

However, because these early results were based on low numbers of exposed people and different histopathological types of brain tumours, no firm conclusions could be drawn. Furthermore, this first study did not include the use of cordless phones (8,10). The next study from the Hardell group included cases diagnosed in the period 1997–2003, and was larger than the first study. This time, the use of cordless phones was also assessed. Further details may be found in the various publications that are based on the results from these studies (11–16).

The Interphone study was conducted at 16 research centres in 13 countries during varying time periods between 2000 and 2004. It was an international collaboration on brain tumour risk and mobile phone use, conducted under the aegis of IARC. Cases were diagnosed during 2000–2004, with slight variations in the different study regions (3,17). In contrast to the Hardell group studies, Interphone did not assess or present results for cordless phone use. These are the only studies to date that provide results for latency periods exceeding 10 years.

Exponential increases in access to and ownership of wireless phones in most countries has occurred since the end of the 1990s. Because the technology is relatively recent, results on health risks for long-term use, exceeding decades, are still lacking. Moreover, in Sweden the major increase in use (duration in minutes of calls) and exposure to radiation fields from these phones (not merely access to or ownership of) in the general population is most evident after 2003 (18).

To obtain results for longer exposure periods of wireless phone use, we conducted an entirely new study on brain tumours. In this article, we present the most recent results for malignant brain tumours. Updated results and discussions of this research area can be found elsewhere (5,19). The study was approved by the ethics committee: Regional Ethics Committee, Uppsala University; Uppsala, Sweden. DNR 2005:367.

## Materials and methods

### Case ascertainment

Sweden comprises six administrative medical regions each having a cancer registry; annually, these registries are linked to the national Swedish cancer register. The reporting to us of newly diagnosed brain tumour cases varied between these six regions, from once a month to once a year from one region (Umeå). In our previous studies covering the time period 1997–2003, we received reports on new cases as these arose, or one to two times per month. For logistical reasons, this was not possible in the present study for the different cancer registries.

### Inclusion criteria

The inclusion criteria specified both men and women aged 18–75 years at the time of brain tumour diagnosis (ICD-7 code 193.0) during the period 2007 to 2009. Furthermore, the diagnosis had to be verified histopathology for all cases and only living cases were included in the study. The cases were reported to us from population-based cancer registries from across all regions of Sweden. For administrative reasons, the Gothenburg region could be included for only the years 2008 and 2009. All patients, both with a malignant or a benign brain tumour, were included in the whole study. Once the inclusion criteria were satisfied, the attending physician was contacted for permission to include the case in the study. The present publication presents results for cases with a malignant brain tumour.

The Swedish Population Registry was used for identification of controls. One control matched on gender and in 5-year age groups was used for each case, both malignant and benign brain tumour cases. All controls were recruited from the same source population (residential) as the cases. Controls were only selected to the finally included living cases. They were assigned the same year as the diagnosis of the respective case as the cut-off in assessing exposure. Thus, the same methods were used as in our previous studies (12,13).

### Exposure assessment

Use of wireless phones, both mobile and cordless, was assessed by a self-administered questionnaire supplemented over the phone. Both cases and controls received an introduction letter and were asked if they were willing to participate and answer the included questionnaire. To get as high response rate as possible two reminders were sent. All mobile phones in Sweden have had either prefix 010 (analogue type) or prefix 07 (digital type). Thus by asking for the prefix it was possible both to verify use of a mobile phone and the type. The questionnaire also contained a number of other questions on, for example, occupational history, exposure to different agents, smoking habits, medical history including hereditary risk factors, and exposure to ionizing radiation. All questions were supplemented over the phone by the interviewer at the same time. A structured protocol was used for all questions as a prompt. The written questionnaire was evaluated and further interviews were made according to the protocol. Most subjects were also phone interviewed to clarify different aspects in the questionnaire. There was no difference regarding supplementary interviews according to being a case (75% supplemented) or a control (70% supplemented). Adjusting for whether or not a supplementary interview was performed did not change the results of the logistic regression analysis.

The ear that had mostly been used during calls with mobile and/or cordless phones was assessed by separate questions; >50% of the time for one side, or equally much for both sides. After informed consent from the patients, medical records including computer tomography (CT) and/or magnetic resonance imaging (MRI) were used to define tumour localization. The matched control was assigned the same side as the tumour of the respective case using the same method as in previous studies (3,12,13,17). The whole procedure was blind to exposure status. Use of the wireless phone was defined as ipsilateral (≥50% of the time), or contralateral (<50% of the time) in relation to tumour side.

All questionnaires received a unique identity number that did not indicate case or control status. Thus, the interviewer was blind to case or control status throughout data processing. The interviewers used a structured protocol that avoided questions that could reveal if the interviewee was a case or a control. All information was coded and entered into a database. A random sample of the questionnaires was coded twice by two independent persons with similar results. Being a case or control was revealed only during the statistical analyses.

### Statistical methods

All analyses were done using StataSE 12.1. Odds ratios (OR) and 95% confidence intervals (CI) were calculated using unconditional logistic regression analysis including the whole control sample (i.e. matched to both malignant and benign cases) to increase the power in the study. This was possible since adjustment/stratification was made for the two matching variables (gender, and age within 5 years).

The unexposed category consisted of people who reported no use of mobile or cordless phones, or a latency period ≤1 year (amount of time between first use of the phone and year of diagnosis). As noted earlier, the same year as for each case diagnosis was used for the corresponding control as the cut-off for exposure accumulation. Furthermore, because of the low number of unexposed cases, a further criterion was used, i.e. regardless of latency being ≤1 year, cumulative use ≤39 h (3rd percentile) of wireless phones in total among the controls was also used as cut-off for the referent group of ‘no exposure’ among cases and controls. The 3rd percentile was chosen to approximately correspond to one working week.

A latency period ≤1 year was used, as in our previous studies, to make it possible to analyse a late effect (promotion) in brain tumour genesis (12,13). Note that latency (time since first use until date of diagnosis) was calculated separately for the respective phone type or combination of phones that were analysed.

Latency was analysed using six time periods, >1–5 years, >5–10 years, >10–15 years, >15–20 years, >20–25 years and >25 years. Cumulative use of the phone types was analysed in quartiles based on use of wireless phones in total among the controls (first quartile >39–405 h, second quartile 406–1,091 h, third quartile 1,092–2,376 h, fourth quartile >2,376 h). Wald's test was performed to analyze the trend of the ORs across the quartiles of the phone types. Latency and cumulative use were also analysed as continuous variables (per year of latency, per 100 h cumulative use) to further explore the dose-response relations.

Adjustment was made for the matching variables gender, age (as a continuous variable) and year of diagnosis. In addition, adjustment was made for socio-economic index (SEI) divided into four categories (blue-collar worker, white-collar worker, self-employed, no work). Note that laterality of the tumour was not available for all cases, e.g., for midline tumours, or for tumours in both hemispheres (n=38). These were dropped from the laterality analysis together with controls (n=306) matched to cases without laterality data in the whole material. Laterality analysis was not made for the whole group of wireless phone users since the side differed for mobile phone and cordless phone for some of the included persons using both phone types (8.3% of the cases, 8.9% of the controls).

Restricted cubic splines were used to visualize the relationship between cumulative use and latency of wireless phones and malignant brain tumours. Adjustment was made for the same variables as in the logistic regression. Four knots were used at the 5th, 35th, 65th and 95th percentiles as suggested by Harrell (20). A p-value for non-linearity was estimated by testing if the coefficient of the second and third spline was equal to zero (20).

Most of the participating cases with a benign brain tumour (n=814) had meningioma (n=709). These results will be presented in another publication. As a further step to evaluate potential recall or observational bias the meningioma cases in the same study were used as the reference entity to the cases with malignant brain tumour, c.f. Hardell (21).

## Results

In Table I, the number of reported malignant cases from the regional cancer registries is shown. The largest numbers of cases excluded from the study were those who were ‘deceased’ (n=520), mostly with an astrocytoma WHO grade IV (glioblastoma multiforme). The implications of this exclusion are addressed below in the discussion section. The second largest group excluded was that with ‘no permission from the treating physician’ (n=56). Thus, of the 1,334 cases with a malignant tumour, 683 (51%) remained eligible for inclusion. Regarding cases with a benign brain tumour (n=920) these results are presented in separate articles; one on meningioma (22) one on acoustic neuroma (23).

Medical records and reports to the cancer registries were used to classify tumour histopathology. Of the 683 cases of malignancy, 593 (87%) answered the questionnaire; 350 were men and 243 women. In Table II, the various diagnoses of malignant brain tumours are shown. Most of the cases were diagnosed with a glioma (astrocytoma, oligodendroglioma, other/mixed glioma; n=546; 92%) with astrocytoma being the most common subtype (n=415; 76% of glioma).

For the total sample of 1,601 cases, an equal number of matched controls received a questionnaire. Note that one case had two tumours, astrocytoma grade IV and meningioma and another case had ependymoma and acoustic neuroma. Of the included controls, 1,368 (85%) answered the questionnaire, 564 were men and 804 women. The mean age was 52 years for cases with malignant brain tumour (median 55, range 18–75) and 55 years for all controls (median 58, range 19–75). Of the cases with meningioma 200 were men and 509 were women. The mean age was 57 years (median 59, range 23–75 years).

In Table III, the results are shown for all malignant brain tumours and use of wireless phones. Analogue phones yielded OR=1.8, 95% CI=1.04–3.3 increasing to OR=3.3, 95% CI=1.6–6.9 in the latency group of >25 years. Note that the latency time was counted from the first use of the specific telephone type; for instance, a 2G user may have used an analogue phone before.

Use of digital 2G phones gave an overall OR=1.6, 95% CI=0.996–2.7. In the latency group >1–5 years, an OR=1.8, 95% CI=1.01–3.4 was calculated. Lower ORs were obtained in the latency groups >5–10 years and >10–15 years increasing to an OR=2.1, 95% CI=1.2–3.6 with latency >15–20 years, which was the longest latency interval.

The results for digital 3G phones showed highest risk in the >5–10 years latency group, OR=1.6, 95% CI=0.5–4.9. This result was based on low numbers and no long-term users existed since this technology is new. One case and no control reported use of only a 3G phone.

A similar pattern as for digital 2G phones was found for use of cordless phones with increased risk in the shortest latency period, then dropping off and again increasing in the latency group >15–20 years to an OR=2.1, 95% CI=1.2–3.8. Only 6 cases and 13 controls reported use of cordless phone with latency >20–25 years, so these results are less reliable.

In Table III we also display results for all uses of digital phones (2G, 3G and/or cordless phone; ‘digital type’). The pattern of an association was similar to 2G and cordless phones, with a statistically significant increased risk in the shortest latency period, then dropping off and again statistically significant increased risk in the latency group >15–20 years giving an OR=2.2, 95% CI=1.3–3.6.

We further show results for all wireless phone use combined. An increased risk was found overall with an OR=1.7, 95% CI=1.04–2.8, increasing in the shortest latency period >1–5 years to an OR=2.6, 95% CI=1.4–5.0, then decreasing somewhat with increasing latency; but with the highest risk is in the longest latency period >25 years with an OR=3.0, 95% CI=1.5–6.0.

In Table IV results are displayed when patients with meningioma in the same study are used as controls. The results were similar as in Table III using the population based controls. Most ORs were somewhat higher using meningioma cases as controls.

Overall, in Table V, ipsilateral use of analogue phones was associated with a higher risk, OR=2.3, 95% CI=1.2–4.5, than contralateral use, yielding OR=1.4, 95% CI=0.7–2.9. Ipsilateral use of digital 2G phones yielded a higher OR than contralateral use. Mobile phones overall for ipsilateral use, resulted in an OR=1.7, 95% CI=1.01–2.9; and for contralateral use, an OR=1.4, 95% CI=0.8–2.5. Ipsilateral use of cordless phones yielded an OR=1.9, 95% CI=1.1–3.2 compared with an OR=1.6, 95% CI=0.9–2.8 for contralateral use.

Cumulative use of wireless phones was analysed in quartiles based on use of wireless phones in total among the controls, see Table VI. Note that for the various phone types, the cumulative time was counted for use of the specific phone, but for the category ‘mobile phones’ all types of mobile phones were included, and for ‘wireless phones’ also use of cordless phones was included. For all phone types and combinations thereof, the highest ORs were found in the fourth quartile, see Table VI. Thus, for analogue phones, an OR=7.7, 95% CI=2.5–24 (p-trend=0.01) was calculated, although based on low numbers. The digital (2G) phone yielded an OR=3.2, 95% CI=1.8–5.6 (p-trend <0.0001) in the same category. Also, UMTS (3G) resulted in an increased risk with an OR=5.1, 95% CI=0.8–32 (p-trend=0.28); but based on low numbers. The fourth quartile of cumulative cordless phone use yielded an OR=3.1, 95% CI=1.8–5.5 (p-trend <0.0001). Wireless phone use overall resulted in an OR=2.5, 95% CI=1.5–4.2 (p-trend=0.0001) in the fourth quartile with >2,376 h of cumulative use.

The ORs increased to statistically significant per 100 h of cumulative use for all types of phones except for UMTS (3G) with borderline significance, see Table VII. In a multivariate analysis including all phone types (i.e. analogue, 2G, 3G and cordless phone) similar results were found although not statistically significant for analogue phones (OR=1.015, 95% CI=0.996–1.034; data not shown). Wireless phone use increased the risk with an OR=1.009, 95% CI=1.006–1.012 per 100 h of cumulative use, Table VII. The risk increased also per additional year of latency, mostly for analogue phones, OR=1.044, 95% CI=1.019–1.070. These results did not change if years of use of any mobile or cordless phone prior to the respective type was included as a covariate in each analysis of the individual phone types (data not shown). Wireless phones overall yielded OR=1.018, 95% CI=1.001–1.036.

In Table VIII, results are presented for malignant brain tumours localized in the temporal lobe or overlapping temporal and adjacent lobe. Higher risk estimates were obtained than for the overall results. Thus, mobile phone use in the latency group >25 years resulted in an OR=4.8, 95% CI=1.7–14 compared with an OR=2.9, 95% CI=1.4–5.8 overall (see Table III for comparison). Cordless phone use in the group with the longest latency, >20–25 years, resulted in an OR=3.3, 95% CI=0.8–14 in the temporal lobe versus an OR=1.5, 95% CI=0.5–4.6 overall, although based on low numbers. Also, for overall wireless phone use, the highest OR was found among those with the longest latency, >25 years, with an OR=5.1, 95% CI=1.8–15 for tumours in the temporal lobe.

In Table IX, results are displayed for use of only one type of wireless phone. Regarding analogue phones, all cases and controls had also used other phone types. Use of only digital 2G types resulted in the highest risk in the shortest latency period >1–5 years with an OR=3.4, 95% CI=1.2–9.5. The risk decreased somewhat with longer latency, but increased again in the longest latency group >15–20 years to an OR=1.8, 95% CI=0.6–4.9. A similar risk pattern was found for use of cordless phones only, with even higher risk estimates, although based on low numbers in the longest latency groups. Use of wireless phones of only the digital type (2G, 3G, cordless phone) yielded an OR=1.7, 95% CI=1.01–2.7 overall, increasing to an OR=2.7, 95% CI=1.4–5.3 in the latency group >1–5 years. A decreased risk was seen with the longer latency period, but, again, it increased with latency >15–20 years to an OR=1.9, 95% CI=1.1–3.4.

Most types of malignant brain tumours were glioma (n=546). Separate analysis of that group of tumours gave similar results as for the whole group of malignant brain tumours. Mobile phone use with latency >25 years resulted in an OR=2.8, 95% CI=1.4–5.7 (data not shown). Also, for cordless phone use, the results were similar as in the overall analysis. Thus, with a latency >15–20 years, an OR=1.9, 95% CI=1.05–3.5 was found.

**Figure 1.**
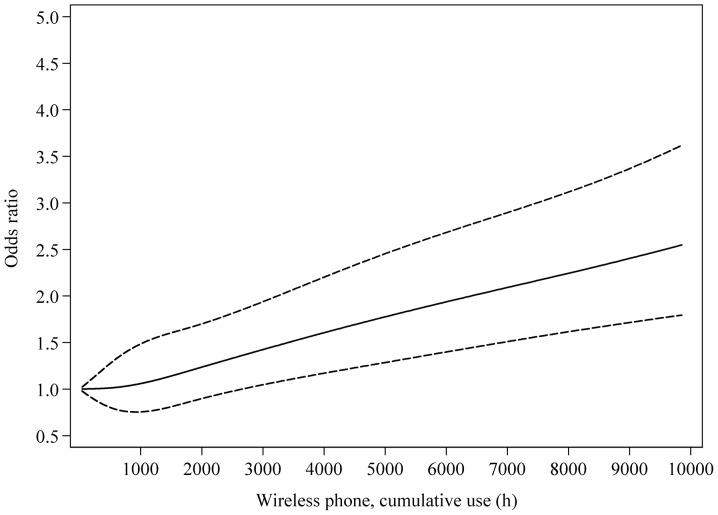
Restricted cubic spline plot of the relationship between cumulative use of wireless phones and malignant brain tumours. The solid line indicates the OR estimate and the broken lines represent the 95% CI. Adjustment was made for age at diagnosis, gender, SEI-code and year of diagnosis. Population based controls were used.

**Figure 2.**
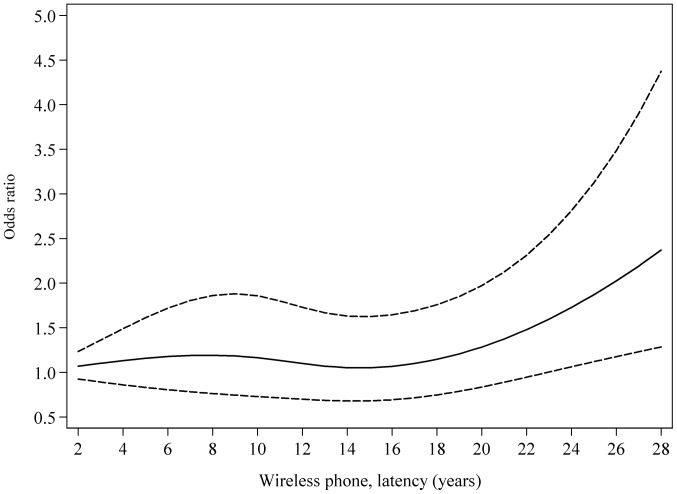
Restricted cubic spline plot of the relationship between latency of wireless phones and malignant brain tumours. The solid line indicates the OR estimate and the broken lines represent the 95% CI. Adjustment was made for age at diagnosis, gender, SEI-code and year of diagnosis. Population based controls were used.

Fig. 1 illustrates the results for cumulative use of wireless phones using the restricted cubic splines method. There was a linear increasing trend of the risk up to 10,000 h (p, non-linearity=0.52). Fig. 2 demonstrates a borderline statistically significant non-linear relationship for the risk and latency using data up to 28 years from first use of a wireless phone before tumour diagnosis (p, non-linearity=0.05). Highest risk was found with longest latency. This finding gives support for RF-EMFs to play a role in the initiation and promotion stages of carcinogenesis.

## Discussion

### Main results and latency (time since first exposure) effects

The main result of this study was a statistically significant increased risk for malignant brain tumours associated with use of wireless phones, OR=1.7, 95% CI=1.04–2.8. The risk increased further in the latency group >1–5 years, but lower ORs were found in the latency groups >5–10 years and >10–15 years. With longer latency periods, the OR increased further with highest risk in the latency group >25 years, OR=3.0, 95% CI=1.5–6.0. From Table III, analogue mobile phones produced a risk increasing with latency, with the highest risk in the latency group >25 years. The OR increased statistically significantly per year of latency, see Table VII. A different pattern was seen for digital wireless phones, both the mobile and cordless types. The risk was higher in the short latency group >1–5 years, then dropped off and increased again with >15 years of latency. Regarding digital 3G mobile phones no conclusions could be drawn. The technique is new and no subject had latency >10 years.

No case or control had used a digital mobile phone with latency >25 years. Only 6 cases and 13 controls had used a cordless phone with latency >20–25 years, so the results for cordless phones with longest latency time were less reliable. Only one case had used only a 3G phone, so firm conclusions about the risk with 3G mobile phone use are not possible from this study. Regarding the use of digital 2G mobile and cordless phones, the OR increased per year of latency with statistically borderline significance. This was explained by the fact that the risk increase was U-shaped in relation to latency period. A further illustration is given in the restricted cubic spline plot showing a borderline statistically significant non-linear relationship, see Fig. 2.

Regarding long-term use of wireless phones and the association with brain tumours, it has not been possible to study longer latency periods than >10 years previously since the technology is too recent. This is the first study to examine effects with a latency time >25 years. This was for analogue phones. Regarding digital 2G mobile phones, the longest duration of latency was >15–20 years. The longest latency for use of cordless phones was >20–25 years with few subjects in that category. The results in this study indicate an early effect in brain tumour genesis seen both for analogue and digital phones, an initiator. Regarding digital phones, we found also a late effect in tumour development, a promoter.

Of interest is that we found that the risk was elevated among those who reported using only digital 2G mobile phones and only cordless phone, see Table IX. The risk was even higher for the use of only cordless phones, a fact that is of importance since all studies other than those from the Hardell group have not paid attention to such use. Including the use of cordless phones in the ‘unexposed group’ would have biased risk estimates towards unity, as discussed elsewhere (4,5).

### Cumulative use

Cumulative use of wireless phones in our present study was divided into quartiles based on cumulative use of wireless phones overall among controls. For all phone types, the highest risk was found in the fourth quartile >2,376 h of cumulative use. This corresponds to about 40 min of wireless phone use per day for 10 years. There was a statistically significant trend for the different phone types, mobile phone use overall, and wireless phones overall. An especially elevated OR was calculated for analogue phone use, OR=7.7, 95% CI=2.5–24, in the fourth quartile. Also, 3G mobile phone use resulted in increasing risk, highest in the fourth quartile, but based on low numbers and no statistically significant trend (p=0.28). These results are also reflected in Table VII, with statistically significant increasing risk per 100 h of cumulative use for all phone types, except for 3G with borderline statistical significance. A linear relationship between cumulative use of wireless phones and the risk for malignant brain tumours is given in Fig. 1.

### Consistency with our previous research

Clearly, digital mobile and cordless phones increase the risk of malignant brain tumours in our present study, as well as in our previous studies. For use of digital type wireless phones only, we found an OR=1.7, 95% CI=1.01–2.7. This finding is consistent with our previous result for the study period 1997–2003. Use of digital mobile and cordless phones yielded an OR=1.4, 95% CI=1.1–1.8 in that study (13). Further analysis in our previous study on use of only mobile phones yielded for glioma increased risk in the >10 year latency group, OR=2.6, 95% CI=1.7–4.1. For use of only cordless phones, an increased risk was found in the >5–10 years latency group, OR=1.9, 95% CI=1.3–2.9, whereas the result for >10 year latency was based on rather small numbers (5,15).

Furthermore, it should be noted that for the study period 1997–2003, we found an increased risk of malignant brain tumours in the latency period >5–10 years for users of wireless phones of the digital type. Thus, digital 2G phones yielded an OR=1.7, 95% CI=1.2–2.2, and for cordless phones, an OR=1.5, 95% CI=1.1–2.0 in that latency group (13). These risks increased further in the latency group >10 years, which was the longest time of wireless phone use in that study. This pattern was different for use of analogue phones, with statistically significant risk only in the group with a latency >10 years, giving an OR=2.4, 95% CI=1.6–3.4, a similar finding to that in the present study.

In summary, our results are consistent with an early effect in carcinogenesis (initiator) by analogue mobile phones, and both an early (initiator) and late (promoter) effect by wireless phones of the digital type.

### Comparison with other studies, e.g. Interphone

In Interphone (data not shown), a statistically significant increased risk for glioma was seen in the group 2–4 years for regular use, with 1–1.9 years use as reference category, OR=1.68, 95% CI=1.16–2.41 (3). The highest OR was found in the 10+ years category for regular use, OR=2.18, 95% CI=1.43–3.31. Results were not presented according to type of mobile phone used. Overall, cumulative use >1,640 h in the shortest latency group of 1–4 years before reference date resulted in an increased risk, OR=3.77, 95% CI=1.25–11.4.

The highest absorption of RF-EMF emissions from a handheld phone is on the same side of the brain (ipsilateral) as the phone is used (9). Highest dose is absorbed in the temporal lobe of the brain. In previous studies, we have found risk being highest for ipsilateral wireless phone use (5,13). In Interphone, cumulative call time of mobile phones >1,640 h, resulted in glioma in the temporal lobe with an OR=1.87, 95% CI=1.09–3.22, and for ipsilateral mobile phone use, an OR=1.96, 95% CI=1.22–3.16 (3). Likewise, in our present study, the OR was higher for ipsilateral use of mobile or cordless phones, see Table V, and for malignant brain tumours in the temporal and overlapping lobes, see Table VIII.

A mean duration of mobile phone use of 2.8 years was reported in a study from USA (24). Overall, no increased risk was found for malignant brain tumours, except for a rare type, neuroepithelioma with OR=2.1, 95% CI=0.9–4.7. The type of mobile phone was not reported. No increased risk for glioma overall or in different groups of duration of regular use, at most >5 years, was reported in another study from USA (25). The type of mobile phone used was not published. An increased risk for glioma with short duration of analogue mobile phone use (1–2 years) was seen in a Finnish study, whereas no increased risk was found for digital phones (26). These results were based on low numbers. Cordless phone use was included in the ‘unexposed’ category in these studies, which is of interest to note since we have found an association with such phone use as reported above.

In a record linkage study from Denmark mobile phone subscribers from January 1, 1982, until December 31, 1995, were identified from the computerized files of the two Danish operating companies, TeleDenmark Mobil and Sonofon, which partly also funded the study. It has produced four articles that we have made a thorough review of (27). We concluded that its many limitations - embedded in the study design from the very beginning and mainly related to poor exposure assessment - cloud the findings of the four reports to such an extent that render them uninformative, at best. The Danish cohort study was included in the IARC evaluation of RF-EMF but the conclusion was that ‘phone provider, as a surrogate for mobile phone use, could have resulted in considerable misclassification in exposure assessment’ (1). Thus, the Danish cohort study is uninformative as to cancer risks from mobile phone use.

### Strengths and limitations

The present study included cases of malignant brain tumours diagnosed during 2007–2009 from across Sweden. For the cases diagnosed during 1997–2003 in our previous study (5), the prevalence of use of mobile phones was highest in the age group 30–54 years for men, and 35–54 years for women. Thus, we included the age group 18–75 years in this study to allow for the longest possible latency time (28). This is in contrast to the Interphone study, which included cases aged 30–59 years. Glioma is the most common malignant brain tumour, and the most common glioma subtype is astrocytoma. Glioblastoma multiforme (WHO grade IV) accounts for 60–75% of all astrocytoma, in this study 66% of the cases with astrocytoma. The peak incidence is between 45–75 years, with a mean age of 61 years and with 80% older than 50 years (29). Thus, limiting the upper age to 59 years for cases as in Interphone (3) would diminish the possibility of finding an increased risk for the long-term use of mobile phones.

Recall and observational bias might be an issue in case-control studies. We investigated in more detail the possibility of that in one of our previous studies (11). Reporting a previous cancer or if a relative helped to fill in the questionnaire did not change the results. Potential observational bias during phone interviews was analysed by comparing the results based on exposure assessment before and after additional phone interviews. The results were similar with no statistically significant differences, showing that our results were unlikely to be explained by observational bias (11).

To further validate exposure in the present study we used meningioma cases as the referents, see Table IV. Thereby the results were similar to those obtained using the population based controls with consistency of the main findings for the main phone types, see Table III. It should be mentioned that a similar method was used previously on the controversy of cancer risks from certain chemicals. Based on clinical observations an increased risk for soft-tissue sarcoma (30) and malignant lymphoma (31) was postulated for exposure to phenoxyacetic acids, chlorophenols and contaminating dioxins. These bed-side observations were followed by case-control studies confirming an association, e.g. Hardell and Sandström (32), Hardell *et al* (33). Using colon cancer cases as referents yielded similar results as when population based controls were used, that is the increased risks were unlikely to be explained by recall or observational bias (21). Thus, similar conclusions can be made in the present study.

In our previous studies, we included only living cases so as to be able to solicit as good an assessment of exposure as possible (10,13). Especially side of head mostly used during phone calls would be difficult to assess using proxy interviews. Excluding deceased cases might, theoretically, bias the results, notably if there is no association between use of wireless phones and brain tumour in that patient group or even a protective effect. We, therefore, did a separate case-control study on deceased cases diagnosed during 1997–2003 with a malignant brain tumour in our previous studies (13) using deceased controls. Relatives of both groups were interviewed and we were able to confirm an increased risk for use of mobile phones (15,34). Thus, inclusion of only living cases and controls in this study would not likely bias the results away from unity.

In total, 1,334 cases were reported from the cancer registries covering all of Sweden. From the Gothenburg region, it was possible to get reports only of cases diagnosed during 2008 and 2009 for administrative reasons. However, exclusion of cases diagnosed during 2007 could not conceivably have biased the results. It has been published that the reporting of new brain tumour cases to the Swedish cancer registry is insufficient (35,36). It is, however, not likely that such omission from our study of not reported cases would be related to the status of being a user or not of wireless phones.

The majority of the cases with a histopathological brain tumour diagnosis that were excluded from this study were deceased (n=520; 39%). As mentioned above we have found an association with use of wireless phones also among the deceased cases (34). Furthermore, for glioma we have found an increased hazard ratio (HR) for survival (37). This was based on all glioma cases, both alive and deceased at the time of the studies as presented in Hardell *et al* (15). An increased hazard ratio was found for >10 years latency for both mobile phone use, HR=1.3, 95% CI=1.0005–1.6, and cordless phone use, HR=1.3, 95% CI=0.9–1.9. HR increased also with the cumulative number of hours of mobile and cordless phone use, with statistically significant trend for tertiles (p=0.01) of use of both phone types. For glioblastoma multiforme (WHO grade IV) use with >10 years latency for mobile phones increased the ratio, HR=1.3, 95% CI=0.9–1.7, and cordless phone, HR=1.8, 95% CI=1.2–2.8, indicating decreased survival for long-term and high cumulative use of wireless phones.

Most of the deceased cases in the present study had a diagnosis of glioblastoma multiforme, WHO grade IV. The median survival in that patient group is less than one year (38). We have reported a higher risk for mobile phone use for high grade glioma (WHO grades III–IV) than for low grade glioma (WHO-grades I–II) (5). Hence, the exclusion of deceased cases with glioblastoma multiforme with poor prognosis in this study might actually have biased the risk estimates towards unity.

We included only cases with a histopathological diagnosis of a brain tumour. We asked the six regional cancer registries not to report cases with only a clinical diagnosis. The reason was that we wanted to insure a confirmed diagnosis of the brain tumour for separate analyses for each tumour type. If necessary, we supplemented the histopathological reports with records from pathology departments around the country after informed consent from the respective case. Thus, we were able to classify all brain tumours based on WHO codes, see Table II. It is not probable that exclusion of cases with only a clinical diagnosis would have biased the results. We checked the Swedish Cancer Registry for the total number of patients with a brain tumour during the study period in the relevant age group. In total, 2,553 patients aged 20–74 years with a brain tumour were reported to the Swedish Cancer Registry versus 2,310 aged 20–74 years with a diagnosis based on histopathological diagnosis in our present study. This is in good agreement with expected numbers since, during 2007–2009, roughly 90% of brain tumour diagnoses in the Swedish Cancer Register were based on histology (http://www.socialstyrelsen.se/statistik/statistikdatabas).

An advantage of this study was the fairly high response rate among both cases and controls. The response rate was 87% (n=593) among the eligible cases. Of the controls, 85% (n=1,368) participated. These response rates were similar to those in our previous studies on malignant brain tumours, 85% (n=1,251) among cases and 84% (n=2,438) among controls (5). Lower response rates were obtained in the Interphone study, namely 64%, range by centre 36–92%, (n=2,765) for glioma cases, and 53%, range 42–74%, (n=7,658) for controls (3). To obtain the most valid results possible, it is always necessary to have the highest possible response rate. In fact, not responding controls in Interphone tended to be less frequent users of mobile phones than participating controls, leading to an underestimation of the risk (4,39,40).

Our study was not designed to include a mini-interview on the use of wireless phones among non-responding cases and controls as done in parts of the Interphone study; we had no ethics clearance for that. Certainly, it would have been of value to verify the use of mobile phones by operator data on the phone traffic. We had no possibility to do this since we did not obtain valid information on the operator used over the years in spite of asking. Furthermore, use of cordless phones, an important source of RF-EMF exposure, is not possible to validate by operator data.

### Statistical considerations

In view of the fact that practically everybody is using a wireless phone of some type today, it is not possible to obtain a large enough ‘unexposed’ group for meaningful statistical calculations. We, therefore, in addition to a latency ≤1 year, used the 3rd percentile (39 h) of cumulative time as a cut-off. Another option to obtain more ‘unexposed’ individuals would have been to change the cut-off for latency. However, doing this would limit the possibility of studying a late effect (promotion) in brain tumour genesis. Furthermore, it is difficult to find users that have been using only one single technology, i.e. NMT, GSM, UMTS, etc. Most users have used several technologies, and those with 3G phones who reported such use may have been unaware that the phone might have been operating on a 2G net for voice, if that was available. The analysis must be viewed with these facts in mind.

In the unconditional logistic regression analysis, all controls, both to cases with malignant and benign brain tumours, were used so as to maximise the statistical power. Analysis using conditional logistic regression yielded overall for wireless phones OR=2.1, 95% CI=1.1–3.7 versus OR=1.7, 95% CI=1.04–2.8 using unconditional logistic regression, see Table III. Using unconditional logistic regression only with controls matched to the malignant cases yielded overall for wireless phones OR=2.0, 95% CI=1.1–3.5. Similar differences were seen for the different phone types i.e. slightly higher risk estimates using conditional logistic regression or unconditional logistic regression with matched controls, although with wider confidence intervals. The latter was due to the fact that only controls matched to malignant cases could be included and also because only discordant matched pairs are considered in a conditional logistic regression analysis. The considerably smaller material would limit the possibility of performing several of the subgroup analyses in this article using this method. Using unconditional logistic regression analysis was possible since adjustment was made for the matching variables of age, gender and year of diagnosis. In addition, adjustment was made for socio-economic index since an association between white-collar work and brain tumours has been reported (41). Not adjusting for any of these variables yielded for wireless phone overall crude OR=2.2, 95% CI=1.4–3.5. No statistically significant interactions were found between the adjustment factors and wireless phone use. In our previous study, we found that heredity and previous X-ray investigations of the head increased the risk for glioma. However, these were independent risk factors with no interaction with use of wireless phones (16). Thus, it was not necessary to adjust for these risk factors in the present study.

More women than men were included as controls. This was because all controls in the study were included in the analysis. Among the cases with benign brain tumour, meningioma was about 2.5 times more common among women than men, an expected number. Thus, adjustment for gender was necessary.

### Biological mechanisms

There is no generally accepted mechanism by which RF-EMF exposure produces changes in DNA. The energy level associated with exposure is too low to cause direct DNA strand breaks and DNA crosslinks. However, DNA damage can be caused by cellular biochemical activities such as free radicals. Several studies indicate that RF-EMFs increase free radical activity in cells (42,43). This process is probably mediated via the Fenton reaction. Hydrogen peroxide is converted into hydroxyl free radicals that are potent cytotoxic molecules. This reaction is catalyzed by iron. High levels of iron are found in metabolic active cells such as cancer cells as well as in cells undergoing abnormal proliferation, but also in brain cells. Glia cells might turn cancerous from DNA damage.

In a recently published study, it was demonstrated that RF-EMF exposure induced the formation of oxidative base damage in a mouse spermatocyte-derived cell line (44). This was mediated by reactive oxygen species (ROS) production. To further elucidate the central role of ROS in RF-EMF exposure-induced DNA base damage, the authors used α-tocopherol pretreatment to antagonize the oxidation of ROS; α-tocopherol is an important lipophilic chain-breaking antioxidant that can inactivate harmful ROS. The protective role of α-tocopherol pretreatment confirmed that ROS are involved in RF-EMF exposure-induced DNA base damage (44). These findings support the idea that low energy RF-EMF that is insufficient to directly induce DNA strand breaks may nonetheless produce genotoxic effects in the form of DNA base damage.

We know little about the earliest events in the genesis of glioma in humans for obvious reasons. However, progression of glioma has been studied in a large series of tumours of different malignancy grades. Patients with low-grade glioma have been followed with later progression to high-grade glioma (45). Thus, since the natural history of most glioma cases, from earliest events to clinical manifestation, is unknown but, most likely requires several decades, the exposure duration has in most studies been incompatible with a tumour initiating effect. This is the first study with long-term use of wireless phones. Interestingly, the most elevated OR was found in the latency group >25 years use. We also found results indicating a late effect on tumour development (promotion).

Initiation and promotion have different effects on the incidence of brain tumours. An initiating effect would have the most direct effect on the incidence. Our results indicate that such an effect would be apparent after more than a 20-year use of mobile phones, and thus be too early to be found in cancer registries. On the other hand, if the exposure acts as a promoter, this would decrease latency time for already existing tumours, giving a temporary, but not a continuous, increase in incidence. In addition, it must be noted that any such effect on tumour development is limited by the magnitude of the shift of the age-incidence function and its slope for the respective tumour type (28).

In conclusion, this study confirmed previous results of an association between use of mobile and cordless phones and malignant brain tumours. The risk was highest for ipsilateral use and tumours in the temporal lobe. The results are consistent with initiation carcinogenesis for analogue phones, and both initiation and promotion carcinogenesis for digital wireless phones.

## Figures and Tables

**Table I.** Descriptive data on the study sample of malignant brain tumour cases diagnosed between 2007 and 2009.

	Malignant
Reported from cancer registries	1,334
Deceased	520
Wrong diagnosis	18
Diagnosed other years	2
No address available	6
Language problems	2
Not capable to participate	47
No permission from physician	56
Total included	683
Refused to participate	90
Answered the questionnaire	593

**Table II.** Histopathology of all malignant brain tumours.

	Men		Women		Total	
		
Histopathology	n	%	n	%	n	%
Astrocytoma grade I–II	53	15.1	44	18.1	97	16.4
Grade I	6	1.7	5	2.1	11	1.9
Grade II	47	13.4	39	16.0	86	14.5
Astrocytoma grade III–IV	205	58.6	113	46.5	318	53.6
Grade III	30	8.6	15	6.2	45	7.6
Grade IV	175	50.0	98	40.3	273	46.0
Medulloblastoma	3	0.9	2	0.8	5	0.8
Oligodendroglioma	32	9.1	37	15.2	69	11.6
Ependymoma	10	2.9	9	3.7	19	3.2
Other/mixed glioma	39	11.1	23	9.5	62	10.5
Other malignant	8	2.3	15	6.2	23	3.9
All malignant	350		243		593	

**Table III.** Odds ratio (OR) and 95% confidence interval (CI) for malignant brain tumours (n=593).

	Analogue			Digital (2G)			Digital (UMTS, 3G)			Mobile phone, total			Cordless phone			Digital type			Wireless phone		
						
Latency	OR	CI	Ca/Co	OR	CI	Ca/Co	OR	CI	Ca/Co	OR	CI	Ca/Co	OR	CI	Ca/Co	OR	CI	Ca/Co	OR	CI	Ca/Co
Total, >1 year	1.8	1.04–3.3	144/260	1.6	0.996–2.7	546/1,208	1.2	0.6–2.4	67/140	1.6	0.99–2.7	548/1,217	1.7	1.1–2.9	461/1,015	1.7	1.04–2.8	571/1,261	1.7	1.04–2.8	571/1,261
>1–5 years	-		0/0	1.8	1.01–3.4	42/109	1.2	0.6–2.4	55/126	1.8	1.002–3.4	41/108	2.0	1.1–3.4	102/209	2.6	1.4–4.9	33/63	2.6	1.4–5.0	32/61
>5–10 years	0.6	0.1–3.1	2/10	1.6	0.97–2.7	213/477	1.6	0.5–4.9	12/14	1.7	0.98–2.8	190/423	1.6	0.95–2.7	188/436	1.6	0.9–2.7	177/421	1.6	0.98–2.8	163/378
>10–15 years	1.4	0.7–3.0	25/51	1.3	0.8–2.2	187/453	-		0/0	1.3	0.8–2.2	163/399	1.6	0.9–2.8	108/248	1.4	0.8–2.3	212/523	1.3	0.8–2.2	184/466
>15–20 years	1.4	0.7–2.7	39/86	2.1	1.2–3.6	104/169	-		0/0	1.5	0.8–2.6	76/174	2.1	1.2–3.8	57/109	2.2	1.3–3.6	143/241	1.7	1.02–3.0	110/231
>20–25 years	2.1	1.1–4.0	48/80	-		0/0	-		0/0	1.9	1.1–3.5	48/80	1.5	0.5–4.6	6/13	1.5	0.5–4.6	6/13	1.9	1.04–3.4	52/92
>25 years	3.3	1.6–6.9	30/33	-		0/0	-		0/0	2.9	1.4–5.8	30/33	-		0/0	-		0/0	3.0	1.5–6.0	30/33

Unexposed latency ≤ 1 year; wireless phone use ≤ 39 h (3rd percentile). Number of exposed cases (Ca) and population based controls (Co) are given. Adjustment was made for age at diagnosis, gender, SEI-code and year of diagnosis.

**Table IV.** Odds ratio (OR) and 95 % confidence interval (CI) for malignant brain tumours (n=593) and meningioma cases (n=708) as reference entity.

	Analogue			Digital (2G)			Digital (UMTS, 3G)			Mobile phone, total			Cordless phone			Digital type			Wireless phone		
						
Latency	OR	CI	Ca/Co	OR	CI	Ca/Co	OR	CI	Ca/Co	OR	CI	Ca/Co	OR	CI	Ca/Co	OR	CI	Ca/Co	OR	CI	Ca/Co
Total, >1 year	2.2	1.1–4.1	144/108	1.8	1.1–3.2	545/592	2.3	0.9–5.7	67/47	1.8	1.1–3.2	547/593	1.8	1.03–3.1	460/521	1.8	1.1–3.1	570/640	1.8	1.1–3.1	570/640
>1–5 years	-		0/0	1.7	0.9–3.4	42/70	2.4	0.96–6.1	55/40	1.7	0.9–3.4	41/69	2.0	1.1–3.7	102/109	2.1	1.05–4.3	33/43	2.1	1.04–4.3	32/42
>5–10 years	1.1	0.1–8.3	2/3	2.0	1.1–3.5	212/235	1.4	0.3–6.0	12/7	1.9	1.1–3.4	189/216	1.7	0.96–3.0	187/216	1.8	1.05–3.2	176/221	1.9	1.05–3.3	162/205
>10–15 years	2.0	0.8–4.9	25/21	1.5	0.9–2.7	187/212	-		0/0	1.5	0.8–2.7	163/185	1.6	0.9–2.8	108/128	1.5	0.9–2.7	212/248	1.4	0.8–2.5	184/226
>15–20 years	1.8	0.8–3.7	39/39	2.3	1.2–4.3	104/75	-		0/0	1.8	0.9–3.3	76/78	2.1	1.1–4.1	57/61	2.2	1.2–3.9	143/121	1.9	1.1–3.4	110/115
>20–25 years	2.4	1.1–5.2	48/29	-		0/0	-		0/0	2.5	1.2–5.2	48/29	1.0	0.3–3.6	6/7	1.1	0.3–3.8	6/7	2.1	1.05–4.2	52/36
>25 years	3.0	1.3–7.4	30/16	-		0/0	-		0/0	3.1	1.3–7.1	30/16	-		0/0	-		0/0	3.1	1.3–7.0	30/16

Unexposed latency ≤ 1 year; wireless phone use ≤ 39 h (3rd percentile). Number of exposed cases (Ca) and controls (Co) are given. One subject with both a malignant brain tumor and a meningioma was excluded from the analysis. Adjustment was made for age at diagnosis, gender, SEI-code and year of diagnosis.

**Table V.** Odds ratio (OR) and 95 % confidence interval (CI) for malignant brain tumours, total, ipsilateral and contralateral exposure.

	All			Ipsilateral			Contralateral		
		
	Ca/Co	OR	95% CI	Ca/Co	OR	95% CI	Ca/Co	OR	95% CI
Analogue	144/260	1.8	1.04–3.3	84/118	2.3	1.2–4.5	46/84	1.4	0.7–2.9
Digital (2G)	546/1,208	1.6	0.996–2.7	322/530	1.7	1.02–2.9	190/404	1.4	0.8–2.5
Digital (UMTS, 3G)	67/140	1.2	0.6–2.4	38/69	1.2	0.5–2.8	24/45	1.1	0.4–3.1
Mobile phone, total	548/1,217	1.6	0.99–2.7	324/534	1.7	1.01–2.9	190/407	1.4	0.8–2.5
Cordless phone	461/1,015	1.7	1.1–2.9	272/454	1.9	1.1–3.2	156/327	1.6	0.9–2.8

Ipsilateral, ≥ 50% use of the phone on the same side as the tumour was located. Contralateral, <50 % use of the phone on the same side as the tumour was located. Tumor laterality not available for 38 cases and 306 controls. Number of exposed cases (Ca) and population based controls (Co) for ever use of the phone type according to exposure criteria are displayed. Note that the subjects could have used more than one phone type. Adjustment was made for age at diagnosis, gender, SEI-code and year of diagnosis.

**Table VI.** Malignant brain tumours (n=593).

	Analogue			Digital (2G)			Digital (UMTS, 3G)			Mobile phone, total			Cordless phone			Digital type			Wireless phone		
						
Quartile	OR	CI	Ca/Co	OR	CI	Ca/Co	OR	CI	Ca/Co	OR	CI	Ca/Co	OR	CI	Ca/Co	OR	CI	Ca/Co	OR	CI	Ca/Co
First	1.7	0.9–3.0	90/184	1.4	0.8–2.3	202/620	1.1	0.5–2.4	35/87	1.4	0.8–2.3	190/587	1.3	0.8–2.2	164/434	1.5	0.9–2.5	113/327	1.5	0.9–2.5	108/317
Second	1.6	0.8–3.4	22/47	1.9	1.1–3.3	138/260	1.0	0.4–2.6	16/34	1.7	1.02–3.0	126/261	1.7	1.01–3.0	120/278	1.4	0.8–2.4	113/320	1.4	0.8–2.4	110/314
Third	2.6	1.2–6.0	18/23	1.4	0.8–2.5	84/199	1.7	0.6–4.8	11/17	1.5	0.9–2.7	95/210	2.1	1.2–3.7	98/194	1.7	1.01–2.9	139/317	1.7	1.003–2.9	137/315
Fourth	7.7	2.5–24	14/6	3.2	1.8–5.6	122/129	5.1	0.8–32	5/2	2.8	1.6–4.8	137/159	3.1	1.8–5.5	79/109	2.6	1.5–4.3	206/297	2.5	1.5–4.2	216/315

Odds ratio (OR) and 95% confidence interval (CI) for cumulative use of wireless phones in quartiles based on use of wireless phones among controls in total. Adjustment was made for age at diagnosis, gender, SEI-code and year of diagnosis. Population based controls were used. First quartile >39–405 h; second quartile 406–1,091 h; third quartile 1,092–2,376 h; fourth quartile >2,376 h. p-trend (Wald's test): analogue, p=0.01; digital (2G), p<0.0001; digital (UMTS, 3G), p=0.28; mobile phone, total, p=0.0001; cordless phone, p<0.0001; digital type, p<0.0001; wireless phone, p=0.0001.

**Table VII.** Odds ratio (OR) and 95% confidence interval (CI) for malignant brain tumours per 100 h cumulative use and per year of latency.

	Per 100 h cumulative use		Per year of latency	
	
	OR	95% CI	OR	95% CI
Analogue	1.037	1.014–1.060	1.044	1.019–1.070
Digital (2G)	1.012	1.007–1.017	1.013	0.989–1.037
Digital (UMTS, 3G)	1.031	0.988–1.076	1.043	0.894–1.216
Mobile phone, total	1.011	1.006–1.015	1.016	0.999–1.034
Cordless phone	1.013	1.007–1.020	1.014	0.992–1.036
Digital type	1.010	1.006–1.013	1.016	0.994–1.039
Wireless phone	1.009	1.006–1.012	1.018	1.001–1.036

Adjustment was made for age at diagnosis, gender, SEI-code and year of diagnosis. Population based controls were used.

**Table VIII.** Odds ratio (OR) and 95% confidenceinterval (CI) for malignant brain tumours located in temporal (n=161) and overlapping lobes [temporofrontal (n=31), temporoparietal (n=22), temporooccipital (n=13)]; in total n=227.

	Analogue			Digital (2G)			Digital (UMTS, 3G)			Mobile phone, total			Cordless phone			Digital type			Wireless phone		
						
Latency	OR	CI	Ca/Co	OR	CI	Ca/Co	OR	CI	Ca/Co	OR	CI	Ca/Co	OR	CI	Ca/Co	OR	CI	Ca/Co	OR	CI	Ca/Co
Total, >1 year	2.4	0.9–6.1	67/260	2.4	0.99–5.6	211/1,208	1.7	0.5–5.9	17/140	2.3	0.99–5.6	212/1,217	2.5	1.04–6.0	175/1,015	2.5	1.05–5.9	221/1,261	2.5	1.05–5.9	221/1,261
>1–5 years	-		0/0	3.3	1.2–8.7	19/109	1.6	0.5–5.9	14/126	3.1	1.2–8.4	18/108	3.0	1.2–7.6	41/209	4.4	1.6–12	15/64	4.5	1.6–13	14/61
>5–10 years	0.9	0.1–9.1	1/10	2.4	0.96–5.7	79/477	2.1	0.3–14	3/14	2.4	0.97–5.8	69/423	2.2	0.9–5.4	68/436	2.4	0.99–5.9	68/420	2.4	0.98–5.9	60/378
>10–15 years	1.6	0.5–5.3	11/51	1.8	0.7–4.3	69/453	-		0/0	1.6	0.7–4.1	57/399	2.3	0.9–5.7	41/248	1.8	0.8–4.5	77/523	1.8	0.7–4.4	66/466
>15–20 years	1.7	0.6–5.0	18/86	3.0	1.2–7.4	44/169	-		0/0	2.0	0.8–5.2	31/174	2.8	1.05–7.4	21/109	3.0	1.2–7.4	57/241	2.3	0.9–5.8	42/231
>20–25 years	2.6	0.9–7.2	21/80	-		0/0	-		0/0	2.7	1.02–7.3	21/80	3.3	0.8–14	4/13	3.4	0.8–14	4/13	2.7	1.04–7.2	23/92
>25 years	5.1	1.7–16	16/33	-		0/0	-		0/0	4.8	1.7–14	16/33	-		0/0	-		0/0	5.1	1.8–15	16/33

Numbers of exposed cases (Ca) and controls (Co) are given. Adjustment was made for age at diagnosis, gender, SEI-code and year of diagnosis. Population based controls were used.

**Table IX.** Odds ratio (OR) and 95% confidence interval (CI) for malignant brain tumours (n=593)

	Analogue only			Digital (2G) only			Digital (UMTS, 3G) only			Cordless phone only			Digital type only		
				
Latency	OR	CI	Ca/Co	OR	CI	Ca/Co	OR	CI	Ca/Co	OR	CI	Ca/Co	OR	CI	Ca/Co
Total, >1 year	-		0/0	1.6	0.9–2.9	78/176	-		1/0	3.5	1.6–7.8	23/44	1.7	1.01–2.7	427/1,001
>1–5 years	-		0/0	3.4	1.2–9.5	9/13	-		1/0	5.8	2.0–17	10/14	2.7	1.4–5.3	32/61
>5–10 years	-		0/0	1.6	0.8–3.2	33/79	-		0/0	3.7	1.3–11	9/19	1.7	1.03–3.0	162/370
>10–15 years	-		0/0	1.3	0.6–2.6	28/68	-		0/0	2.0	0.4–9.4	3/8	1.3	0.8–2.2	163/418
>15–20 years	-		0/0	1.8	0.6–4.9	8/16	-		0/0	2.9	0.2–39	1/2	1.9	1.1–3.4	68/140
>20–25 years	-		0/0	-		0/0	-		0/0	-		0/1	0.6	0.1–2.7	2/12
>25 years	-		0/0	-		0/0	-		0/0	-		0/0	-		0/0

Number of exposed cases (Ca) and population based controls (Co) are given. Results are given for use of only a specific phone type or use of both mobile and cordless phones. Adjustment was made for age at diagnosis, gender, SEI-code and year of diagnosis.
